# Fire Proofing Clothing

**Published:** 1915-11

**Authors:** 


**Fire Proofing Clothing.** Chas. F. Pabst of Brooklyn, Western Med. Times, July, states that ammonium phosphate, 1 ℔ to 1 gal. of water is efficient and does not injure garments. The garments are soaked for 5 minutes and allowed to dry.

